# Environmental Enrichment Enhances Ca_v_ 2.1 Channel-Mediated Presynaptic Plasticity in Hypoxic–Ischemic Encephalopathy

**DOI:** 10.3390/ijms22073414

**Published:** 2021-03-26

**Authors:** Suk-Young Song, Soonil Pyo, Sungchul Choi, Hee Sang Oh, Jung Hwa Seo, Ji Hea Yu, Ahreum Baek, Yoon-Kyum Shin, Hoo Young Lee, Ja Young Choi, Sung-Rae Cho

**Affiliations:** 1 Department and Research Institute of Rehabilitation Medicine, Yonsei University College of Medicine, Seoul 03722, Korea; fgkmn@naver.com (S.-Y.S.); neuro94@yuhs.ac (S.P.); sungchulc@gmail.com (S.C.); tommy698@gmail.com (H.S.O.); zugula@yuhs.ac (J.H.S.); onlyjin112@yuhs.ac (J.H.Y.); ahreumbaek@yonsei.ac.kr (A.B.); kyum309@hanmail.net (Y.-K.S.); 2 Graduate Program of NanoScience and Technology, Yonsei University, Seoul 03722, Korea; 3 Brain Korea 21 Plus Project for Medical Sciences, Yonsei University College of Medicine, Seoul 03722, Korea; 4 Department of Medicine, Yonsei University College of Medicine, Seoul 03722, Korea; 5 Department of Rehabilitation Medicine, Yonsei University Wonju College of Medicine, Wonju 26493, Korea; 6 Department of Medicine, the Graduate School of Yonsei University, Seoul 03722, Korea; raphaellapmr@gmail.com; 7 TBI Rehabilitation Center, National Traffic Injury Rehabilitation Hospital, Yangpyeong 12564, Korea; 8 Department of Rehabilitation Medicine, Seoul National University Hospital, Seoul University College of Medicine, Seoul 03080, Korea; 9 National Traffic Injury Rehabilitation Research Institute, National Traffic Injury Rehabilitation Hospital, Yangpyeong 12564, Korea; 10 Department of Rehabilitation Medicine, Chungnam National University Hospital, Chungnam National University College of Medicine, Daejeon 35015, Korea; jaychoi3399@gmail.com; 11 Rehabilitation Institute of Neuromuscular Disease, Yonsei University College of Medicine, Seoul 03722, Korea

**Keywords:** environmental enrichment, hypoxic–ischemic encephalopathy, calcium channels, synaptic plasticity

## Abstract

Hypoxic–ischemic encephalopathy (HIE) is a devastating neonatal brain condition caused by lack of oxygen and limited blood flow. Environmental enrichment (EE) is a classic paradigm with a complex stimulation of physical, cognitive, and social components. EE can exert neuroplasticity and neuroprotective effects in immature brains. However, the exact mechanism of EE on the chronic condition of HIE remains unclear. HIE was induced by a permanent ligation of the right carotid artery, followed by an 8% O_2_ hypoxic condition for 1 h. At 6 weeks of age, HIE mice were randomly assigned to either standard cages or EE cages. In the behavioral assessments, EE mice showed significantly improved motor performances in rotarod tests, ladder walking tests, and hanging wire tests, compared with HIE control mice. EE mice also significantly enhanced cognitive performances in Y-maze tests. Particularly, EE mice showed a significant increase in Ca_v_ 2.1 (P/Q type) and presynaptic proteins by molecular assessments, and a significant increase of Ca_v_ 2.1 in histological assessments of the cerebral cortex and hippocampus. These results indicate that EE can upregulate the expression of the Ca_v_ 2.1 channel and presynaptic proteins related to the synaptic vesicle cycle and neurotransmitter release, which may be responsible for motor and cognitive improvements in HIE.

## 1. Introduction

Hypoxic–ischemic encephalopathy (HIE) is a brain condition that is caused by a lack of oxygen and limited blood flow in infants [1]. This kind of injury can cause neurological disabilities, including seizures, cerebral palsy, and cognitive and motor dysfunction in infants [2]. Due to their susceptibility, neurons can be permanently damaged when perfusion is halted for merely more than 5 min, ultimately leading to cell apoptosis [3,4]. The outcomes of HIE exist on a spectrum, and the only current therapy for HIE is hypothermia, which has to be initiated within the first 6 h of life, thus making it critical to identify and develop further therapeutic strategies to improve brain function [1,5].

The mouse model for HIE has been developed to model human perinatal HIE, and it can be constructed by the permanent ligation of the common carotid artery (CCA) followed by exposure to a hypoxic condition for a short period of time [6]. This event can induce permanent synapse dysfunction and degeneration in various brain regions [7,8]. Moreover, significant cerebral infarction and malfunction in sensorimotor reflex performance, after HIE injury, were observed in neonatal mice [9,10]

Previous studies have shown that neurons at the penumbra, which are conserved functionally and structurally, are responsible for functional recovery and presynaptic alterations [11,12]. Presynaptic dysfunctions, such as changes in the intracellular level of Ca^2+^ and improper synaptic vesicle cycling, further lead to synaptic failure [13,14]. However, these dysfunctions can be partially rescued by various treatments [15,16].

Environmental enrichment (EE), which consists of complex combinations of physical, cognitive, and social stimuli, is a method of improving rodent welfare [17,18]. EE is also considered the modification of cages that mimics the human exercise/rehabilitation model [19,20]. The beneficial effects of EE on strength, sensorimotor, physiological, and psychological functions in neonatal hypoxic–ischemic (HI) animal models, have been highlighted in recent studies [21,22,23].

Exposure to more enriched cages can induce neuroplasticity, with a higher expression of synaptic proteins, higher rates of synaptogenesis, and more complex dendrite arbors, by increasing physical and social stimuli [18,24]. Neuroplasticity is considered crucial for functional recovery from brain injury in developing brains [25,26,27]. Even for the chronic phase of stroke, the beneficial effects of EE have been highlighted in both preclinical and clinical studies [28,29,30].

Among many presynaptic active zone proteins, Rab3, Munc13, Munc18, SNAP25, syntaxin, VAMP2, and the calcium channel Ca_v_ 2.1, have been reported to affect synaptic plasticity. Rab3 can regulate neurotransmitter exocytosis via its GTP binding property [31,32], and is considered as an essential component for regulating PKA-dependent LTP [33]. Munc13 can induce conformation change of syntaxin upon interaction with the 3a domain of the Munc18-syntaxin complex, resulting in the synthesis of the tetramer of Munc13, Munc18, syntaxin, VAMP2 [34,35], and, with the arrival of SNAP25, the full SNARE complex is assembled as Munc18 is released [36]. From this view, it can be inferred that Munc13 participates in short-term presynaptic plasticity [37], and Munc18 contributes to the improvement of synaptic function probability and plasticity [38,39]. SNAP25, syntaxin, and VAMP form a complex called the SNARE complex, which functions as the main machinery of membrane fusion [40,41,42]. Its role was implicated in the regulation of calcium channels [13], and its effect on neuroregeneration has been identified [43,44].

The P/Q type voltage-dependent calcium channel, Ca_v_ 2.1, is one of the major sources of calcium influx and is responsible for neurotransmitter exocytosis. Its upregulation is known to modify synaptic strength [45], contribute to short-term plasticity [46,47], and contribute to long-term plasticity [48]. These above proteins have noteworthy implications for synaptic plasticity due to their major roles in synaptic transmission. Thus, we looked for presynaptic active zone proteins and calcium channel Cav2.1, mentioned above, to verify whether EE on HIE models enhances neurobehavioral function via inducing neural plasticity.

There is a lack of basic data to support the mechanism underlying EE-mediated neuroplasticity in the chronic condition of HIE. In this study, we asked whether functional improvements and changes induced by EE are accompanied with changes of presynaptic proteins, related to the synaptic vesicle cycle and neurotransmitter release, in various brain regions after HI brain injury.

## 2. Results

### 2.1. EE Improves Motor Coordination and Strength in HIE Mice

**Figure 1:**
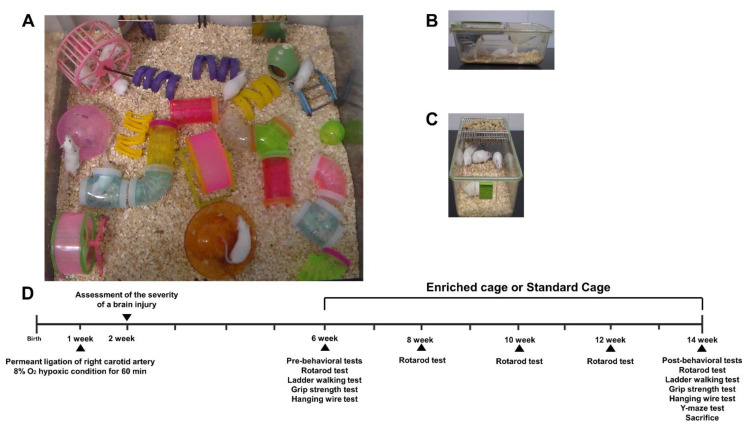
The experimental scheme of this study. (**A**) The representative picture of an environmental enrichment (EE) cage. (**B**,**C**) The representative pictures of standard (control) cages. (**D**) Schematic overview of the experimental design. A total of 60 mild HI mice were selected and randomly separated into 2 groups (control, *N* = 30; EE, *N* = 30), 7 days after surgery based on brain severity, and a total of 15 normal, intact mice were allocated to the standard cages. The location of EE objects was changed once every three days. At 14 weeks of age, all mice were sacrificed for molecular and histological analysis.

HI mice were randomly allocated to either EE cages (Figure 1A) or standard cages (Figure 1B,C) at 6 weeks of age. Behavioral assessments were conducted based on the experimental scheme (Figure 1D).

**Figure 2:**
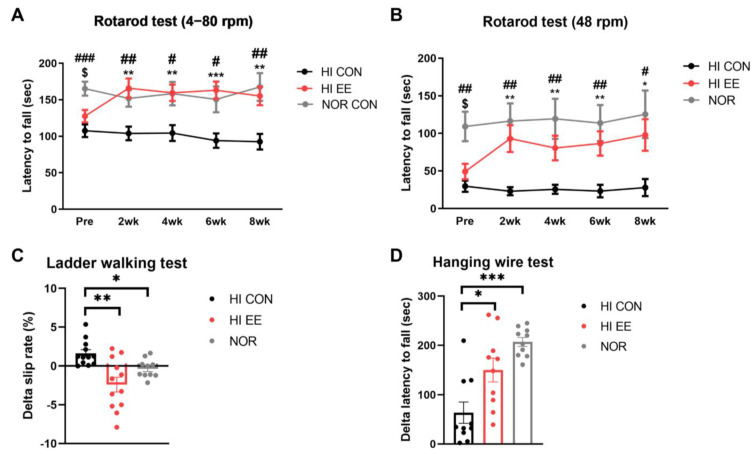
EE improves motor coordination and strength in HIE mice. (**A**) Accelerating rpm rotarod performance (4–80 rpm) at 2-week interval. The HI EE group and the NOR group significantly outperformed the HI control group throughout the condition period (Bonferroni multiple comparisons test). (**B**) Constant rpm rotarod performance (48 rpm) at 2-week interval (Bonferroni multiple comparisons test). The HI EE group and the NOR group significantly outperformed the HI control group throughout the study period. Data are expressed as mean ± SEM with 30 mice for the HI CON and EE groups, and 15 mice for the NOR group. The asterisk (*) indicates a significant difference between the HI CON group and the HI EE group (** *p* < 0.002, *** *p* < 0.0002). The pound sign (#) indicates a significant difference between the HI CON group and the NOR group (^#^ *p* < 0.01, ^##^ *p* < 0.002, ^###^ *p* < 0.0002). The dollar sign ($) indicates a significant difference between the HI EE group and the NOR group (^$^ *p* < 0.01). (**C**) Ladder walking tests were performed at week 6 and week 14. Significant differences in delta left limb slip rate (post–pre) were observed between the HI CON group and the HI EE group, and in the HI CON group and the NOR group, respectively, over the condition period (* *p* < 0.05, ** *p* < 0.01, the least significant difference test). Data are expressed as mean ± SEM with 12 mice for the HI groups and 10 mice for NOR group. (**D**) Hanging wire tests were performed at week 6 and week 14. Significant differences in delta latency to fall (post–pre) were observed between the HI control group and the HI EE group, and in the HI CON group and the NOR group, respectively (* *p* < 0.05, *** *p* < 0.001, the least significant difference test). Data are expressed as mean ± SEM with 10 mice for all groups. HI, hypoxic–ischemic; CON, control; NOR, normal intact.

The HI EE-treated group showed significant improvement and functional recovery in all examined motor function tests. A rotarod test in both accelerating (4–80 rpm, Figure 2A) and constant (48 rpm, Figure 2B) paradigms showed that NOR mice had significantly higher latency to fall than HI CON mice and HI EE mice right before the initiation of the housing condition, respectively (NOR v. HI CON, ^#^ *p* < 0.01, ^##^ *p* < 0.002, ^###^ *p* < 0.0002; NOR v. HI EE, ^$^ *p* < 0.01, ^$$^ *p* < 0.002, ^$$$^ *p* < 0.0002). The differences between HI EE mice and NOR mice was not statistically significant throughout the condition period. The improved motor function of HI EE mice was maintained throughout the condition period in both accelerating and constant paradigms, compared to those of HI control mice (* *p* < 0.01, ** *p* < 0.002, *** *p* < 0.0002). Similarly, the ladder walking test showed that EE mice had a significant reduction in delta (post–pre) left limb slip rate compared to that of HI control mice, and NOR mice compared to that of HI control mice, respectively (* *p* < 0.05, ** *p* < 0.01, Figure 2C). HI EE mice had a significantly higher delta (post–pre) latency to fall compared to that of HI control mice, and NOR mice compared to that of HI control mice, respectively (* *p* < 0.05, *** *p* < 0.001, Figure 2D). 

### 2.2. EE Improves Short-Term Spatial Memory in HIE Mice

**Figure 3:**
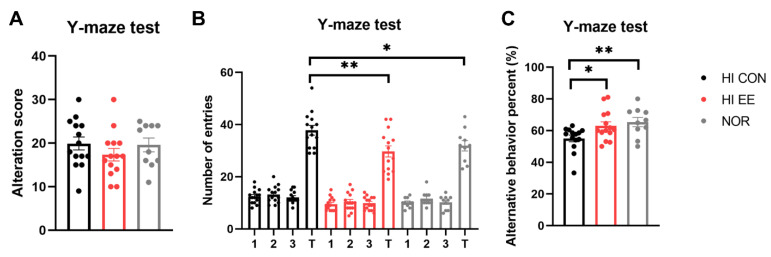
EE improves short-term spatial memory in HIE mice. (**A**) Raw alternation scores of HI control, EE, and NOR mice in the Y-maze. There was no significant difference among the groups in raw alternation scores. (**B**) Number of arm entries in the Y-maze. There was a significant difference in total entries of the HI CON group compared to the HI EE group, and in the HI CON group compared to the NOR group, respectively (** *p* < 0.01, * *p* < 0.05, the least significant difference test). (**C**) Alternation percent in the Y-maze test. A significant increase was observed in the HI EE group compared to the HI control group, and in the NOR group compared to the HI CON group, respectively (* *p* < 0.05, ** *p* < 0.01, the least significant difference test). Data are mean ± SEM with 14 mice for the HI groups and 10 mice for NOR group.

The HI EE-treated group showed significant improvement and functional recovery in cognitive function. Raw scores of the alternative behavior and number of entries are represented in Figure 3A,B, respectively. Although the raw scores were not significantly different among the three groups, the HI EE group and NOR intact group had significantly fewer total entries than the HI control group (** *p* < 0.01, * *p* < 0.05). This result is similar to the maze results of previous studies, and indicates that long-term exposure to EE may decrease levels of anxiety, as indicated by the significantly low number of total entries [49,50]. Overall, HI EE mice and NOR intact mice had a significantly higher alterative behavior percent compared to that of HI control mice (* *p* < 0.05, ** *p* < 0.01, Figure 3C). This result indicates that HI EE mice can retain fine working short-term memory after long-term exposure to EE. 

### 2.3. EE Upregulates the Expression of Ca_v_ 2.1 in the Cerebral Cortex and Hippocampus in HIE Mice

**Figure 4:**
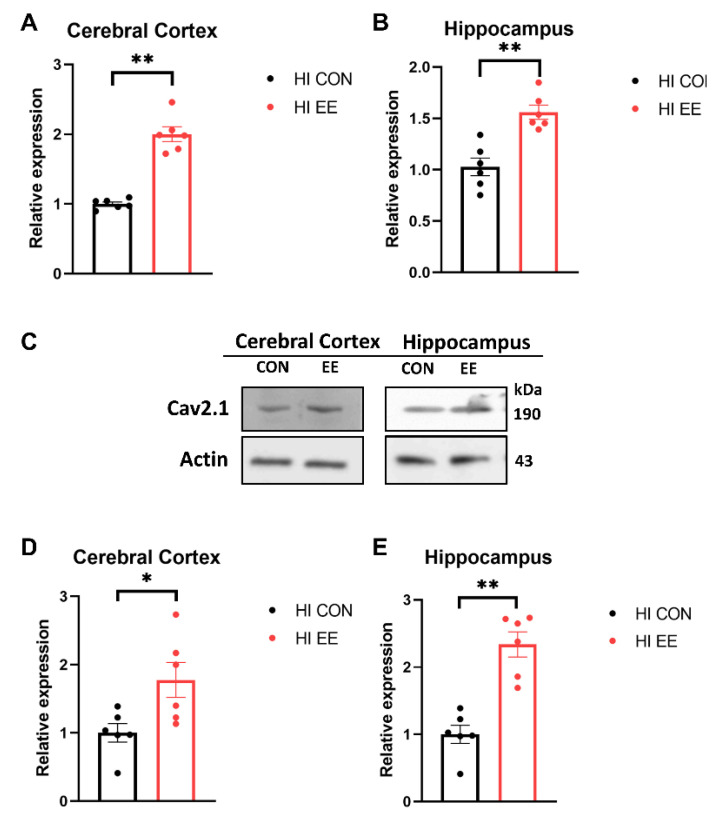
EE significantly increases the expression of Ca_v_ 2.1 in the cerebral cortex and hippocampus in HIE mice. (**A**,**B**) The qRT-PCR results of Ca_v_ 2.1 in the cerebral cortex and hippocampus. A significant difference was observed between HI control mice and HI EE mice in the cerebral cortex and hippocampus. (**C**) The representative Western blot (WB) images of Ca_v_ 2.1 cerebral cortex and hippocampus. (**D**,**E**) A quantification of Ca_v_ 2.1 protein expression in the cerebral cortex and hippocampus. A significant difference was observed between HI control mice and HI EE mice in the cerebral cortex and hippocampus. Molecular data are expressed as mean ± SEM with 6 mice per group (* *p* < 0.05, ** *p* < 0.01, Mann–Whitney U test).

To examine EE-induced changes in gene expression in the synaptic proteins, a qRT-PCR was performed. In our qRT-PCR analysis, EE mice showed a significant increase in the mRNA expression of Ca_v_ 2.1 compared to that of HI control mice in the cerebral cortex (** *p* < 0.01, Figure 4A) and hippocampus (** *p* < 0.01, Figure 4B). To examine EE-induced changes in protein expression in the synaptic proteins, a Western blot (WB) was performed. The representative WB images of the Ca_v_ 2.1 protein are shown in Figure 4C. In WB analysis, EE mice showed a significant increase in the protein expression of Ca_v_ 2.1 compared to that of HI control mice in the cerebral cortex (* *p* < 0.05, Figure 4D) and hippocampus (** *p* < 0.01, Figure 4E).

### 2.4. EE Induces Presynaptic Plasticity through the Higher Colocalization of Ca_v_2.1 with MAP2 in the Cerebral Cortex and Hippocampus in HIE Mice

**Figure 5:**
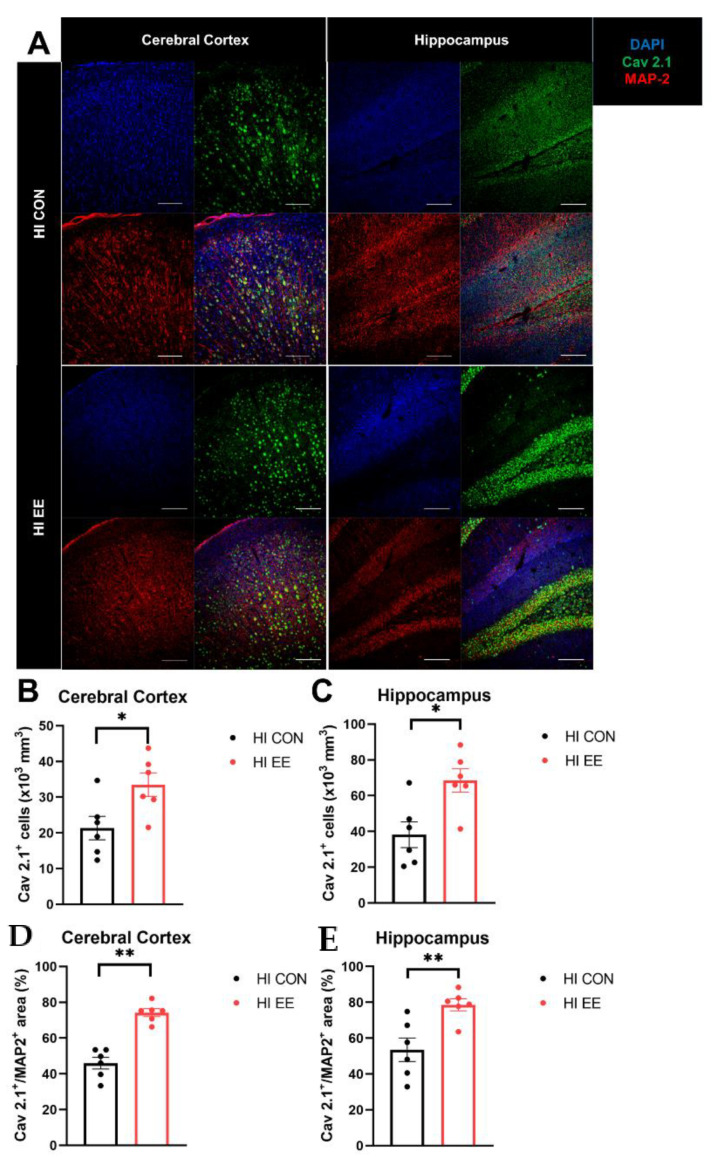
EE mediates presynaptic plasticity through higher colocalization of Ca_v_2.1 with MAP2 in the cerebral cortex and hippocampus in HIE mice. (**A**) The representative confocal images of Ca_v_ 2.1 and MAP2 in the cerebral cortex and hippocampus. A white bar is 100 μm. (**B**,**C**) The number of Ca_v_ 2.1^+^ cells in the cerebral cortex and hippocampus was significantly different between HI control mice and HI EE mice. (**D**,**E**) A significant difference in the area of Ca_v_ 2.1^+^ MAP2^+^ was observed between HI control mice and HI EE mice in the cerebral cortex and hippocampus, respectively. Ca_v_ 2.1, Ca_v_ 2.1 P/Q voltage-dependent calcium channel; MAP2, microtubule associated protein 2, a mature neuronal marker; DAPI, 4′,6-diamidino-2-phenylindole, nuclear staining. Histological data are expressed as mean ± SEM with 6 mice per group (* *p* < 0.05, ** *p* < 0.01, Mann–Whitney U test).

To validate EE-induced changes in the expression of Ca_v_ 2.1 and colocalization with a specific neuronal subtype, immunohistochemistry was performed. The representative confocal images of the Cav 2.1 and MAP2 proteins are shown in Figure 5A. In the immunohistochemistry (IHC) analysis, EE mice had a significantly higher number of Cav 2.1 positive cells in the cerebral cortex (* *p* < 0.05, Figure 5B) and hippocampus (* *p* < 0.05, Figure 5C), and a higher area of Cav 2.1^+^MAP2^+^ cells compared to that of HI control mice in the cerebral cortex (** *p* < 0.01, Figure 5D) and hippocampus (** *p* < 0.01, Figure 5E). 

### 2.5. EE Upregulates the Expression of Presynaptic Proteins in the Cerebral Cortex and Hippocampus in HIE Mice

**Figure 6:**
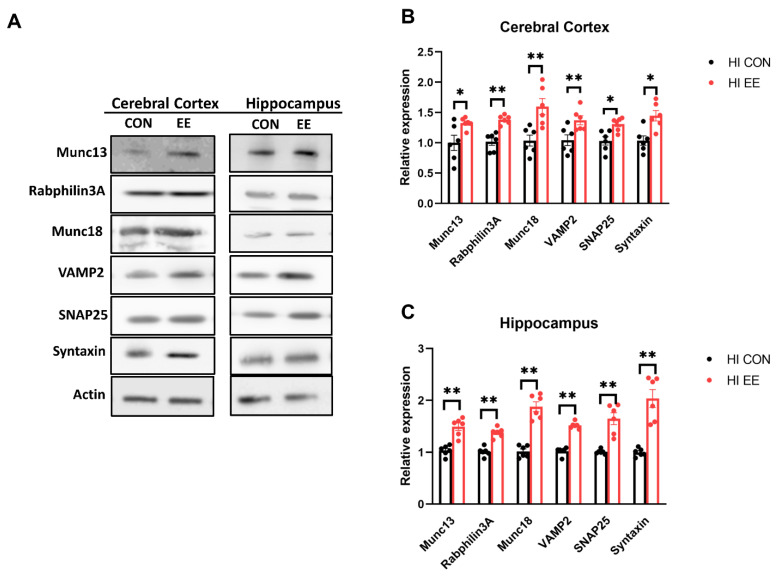
EE upregulates the expression of presynaptic proteins in the cerebral cortex and hippocampus in HIE mice. (**A**) The representative WB images of presynaptic proteins in the cerebral cortex and hippocampus. (**B**) The WB results of presyanptic proteins in the cerebral cortex. A significant difference was observed between HI control mice and HI EE mice in Munc 13, Raphilin3A, Munc18, VAMP2, SNAP25, and Syntaxin. (**C**) The WB results of presyanptic proteins in the hippocampus. A significant difference was observed between HI control mice and HI EE mice in Munc 13, Raphilin3A, Munc18, VAMP2, SNAP25, and Syntaxin. Molecular data are expressed as mean ± SEM with 6 mice per group (* *p* < 0.05, ** *p* < 0.01, Mann–Whitney U test).

The representative WB images of the synaptic protein are shown in Figure 6A. In WB analysis, EE mice showed a significant increase in the protein expression of Munc 13 (* *p* < 0.05), Rabphilin 3A (** *p* < 0.01), Munc 18 (** *p* < 0.01), VAMP2 (** *p* < 0.01), SNAP25 (* *p* < 0.05), and Syntaxin (* *p* < 0.05), compared to those of HI control mice in the cerebral cortex (Figure 6B). EE mice showed a significant increase in Munc 13 (** *p* < 0.01), Rabphilin 3A (** *p* < 0.01), Munc 18 (** *p* < 0.01), VAMP2 (** *p* < 0.01), SNAP25 (** *p* < 0.01), and Syntaxin (** *p* < 0.01), compared to those of HI control mice in the hippocampus (Figure 6C).

## 3. Discussion

HI brain damage in the perinatal period remains one of the main causes of permanent neurodevelopmental impairments and mortality [1]. Our present study provided evidence that exposure to EE, starting 35 days after an HI brain injury, can still improve motor and cognitive deficits to the extent of normal intact mice. Moreover, the molecular and histological analysis also revealed that EE upregulates Ca_v_ 2.1 expression and the presynaptic related proteins in various brain regions, such as the cerebral cortex and hippocampus in HI mice. In addition to these brain regions, we also noticed a significant increase of Ca_v_ 2.1 and a higher area of Ca_v_ 2.1^+^MAP2^+^ cells in the striatum of EE mice compared to that of HI control mice (Figure S1). Moreover, this higher colocalization is only noticed in neuron-related markers, such as MAP2 and NeuN, but not in GFAP, an astrocyte-related marker (Figure S2).

Our WB analysis indicated that the significant upregulation of synaptic proteins is prominent in the hippocampal and the neocortical regions. This may be due to the fact that these areas tend to be more sensitive to treatments and stressors, such as oxidative stress, which has more potential to affect brain plasticity [51,52,53]. HI injury can induce more damage to these brain regions [54], and this injury may be neuroprotected and more neuroplastic by long-term exposure to EE. 

Upregulation in the Ca_v_ 2.1 expression and the presynaptic related proteins may contribute to behavioral improvements in stroke. Previous studies have shown that exposure to EE can improve behavioral functions through synaptic plasticity in intact and stroke models [18,24,55,56,57]. However, despite recent data showing that synaptic plasticity is associated with exercise and behavioral improvement, there are only a few basic studies focusing on the effect of EE on the expression of synaptic proteins in stroke models [16,58]. Our findings further add to these existing literatures by the EE-mediated upregulation of Ca_v_ 2.1 expression and presynaptic related proteins in the cerebral cortex and hippocampus.

Voltage-gated Ca^2+^ (Ca_v_) channels play an important role as the primary mediator of membrane depolarization [59,60]. Massive calcium entry through Ca_v_ channels triggers neuronal firing and neurotransmitter release from synaptic vesicles, which are highly dependent on the physical distance between Ca_v_ 2.1 and synaptic vesicle-related proteins [61,62,63]. Ca_v_ channels can transduce electrical activity into the flow of Ca^2+^ ions that initiate the vesicular release of neurotransmitters at synapses, interacting directly or indirectly with a variety of synaptic proteins in a presynaptic terminal [45,64,65,66].

Studies have shown that the functional disruption of Ca_v_ channels and synaptic loss is accompanied by stroke, and partly reversed by motor rehabilitation with the increased expression of synaptic proteins in the peri-infarct region [67,68]. These previous studies are consistent with our results, in that EE mediated the increased expression of synaptic proteins in the peri-infarct region of the cerebral cortex and hippocampus [68]. Moreover, motor function recovery and motor cortical reorganization can occur at a later stage of stroke through rehabilitative training [69,70,71]. Therefore, boosting this recovery process and enhancing residual brain synapses and networks are critical for better outcomes of stroke patients.

Previous studies have demonstrated the close relationship between motor improvement, synaptic plasticity, and the altered expression of synaptic proteins [72,73,74]. Motor improvement is associated with the increase in the expression of presynaptic proteins [75,76]. Consistent with the previous studies, our results also indicated that the EE-induced increase in the expression of presynaptic-related proteins is associated with motor improvement in HI mice. 

The novelty of our present study is that the expression of Ca_v_ 2.1, and the close interaction between Ca_v_ 2.1 and presynaptic related proteins, may be sensitive to the effects of EE in various brain regions. Moreover, delayed exposure to EE, starting 35 days after HI brain injury, can still be therapeutic in stroke, as indicated by improved behavioral outcomes. The limitation of our study is the strict criterion on subject selection. Mild HI mice (less than 20% of cortical cavity) were only included in this study to obtain visible tissues of the cerebral cortex and hippocampus. Moreover, since our data did not provide compelling evidence on the close relationship between the increased expression of Ca_v_ 2.1, synaptic plasticity, and functional improvement, further studies to investigate the limitation of functional improvements induced by EE using a Ca_v_ 2.1 antagonist are needed.

## 4. Materials and Methods

### 4.1. Ethics Statement and Experimental Animals

All procedures were reviewed and approved by the Association for Assessment and Accreditation of Laboratory Animal Care (AAALAC) (2016) and the Institutional Animal Care and Use Committee (IACUC) of Yonsei University Health System (permit number: 2018-0110). All procedures were in accordance with the guidelines of the National Institutes of Health’s Guide for the Care and Use of Laboratory Animals. These regulations, notifications, and guidelines originated, and were modified, from the Animal Protection Law (2008), the Laboratory Animal Act (2008), and the Eighth Edition of the Guide for the Care and Use of Laboratory Animals (NRC 2011). Mice were provided food and water ad libitum under alternating 12-h light/dark cycles, according to animal protection regulations. They were sacrificed at 8 weeks after the housing conditions, under ketamine (100 mg/kg) and xylazine (10 mg/kg) anesthesia by intraperitoneal injection. All efforts were made to minimize animal suffering.

### 4.2. Construction of Hypoxic–Ischemic Encephalopathy (HIE) Model

At postnatal day 7, HI brain injury was induced by a permanent ligation of the unilateral right common carotid artery, right below where the external and internal carotid arteries branch out; acute exposure to hypoxic condition (8% O_2_, 92% N_2_) was then performed, as previously described [6]. With a visual microscopy, severity of a brain injury was assessed at two weeks of age, and mice whose brain lesion size exceeded 20% of the cortical cavity, on the ipsilateral side of the brain, were excluded in this study.

### 4.3. Experimental Procedures and Cage Condition

At 6 weeks of age, a total of 60 male HI ICR/CD-1 were randomly housed to either standard conditions (SC, *n* = 30) or an enriched environment (EE, *n* = 30) in this study. The condition lasted until 14 weeks of age. EE mice freely accessed novel objects and large-scale social interaction (12~15 mice/cage) (Figure 1A) relative to control mice (5 mice/cage) (Figure 1B,C). After the condition period, all mice were sacrificed for either molecular or histological assessments at 14 weeks of age. The studied brain regions were dissected based on the mouse brain gross anatomy atlas, and the stereotaxic coordinates for the cerebral cortex, hippocampus, and striatum were (ML = −1.0, AP = 0.1, DV = 1.0), (ML = −1.0, AP = −2.0, DV = 2.0), and (ML = −1.0, AP = 0.1, DV = 2.5), respectively. 

### 4.4. Behavioral Assessments

#### 4.4.1. Rotarod Test

A rotarod (No. 47,600; UGO Basile, Comerio, VA, Italy) test was used to evaluate the motor coordination and balance of the experimental mice using an accelerating (4~80 RPM) speed paradigm and a constant (48 RPM) paradigm. After placing mice on the rotating rods, the time taken for the mice to fall from the rods was measured for 300 s [18].

#### 4.4.2. Ladder Walking Test

The ladder walking test can assess subtle disturbances of motor function through qualitative and quantitative analysis of walking [6,77]. This test was performed at five to six weeks of age as a baseline study. The ladder walking test was performed 8 weeks after intervention. In the ladder walking test, mice were required to walk a distance of 1 m, four times, on a horizontal ladder with metal rungs (Jeung Do Bio and Plant Co., Seoul, Korea) located at differing distances apart. The number of slips in each forelimb was measured using videotape analysis. The variance between the control and EE groups was calculated as the difference in the percentage of slips on the transverse rungs of the ladder relative to the total number of steps taken by each forelimb of the EE mice compared that of the controls.

#### 4.4.3. Hanging Wire Test

The hanging wire test evaluated neuromuscular strength of the paws of the experimental mice [78]. To this end, mice were suspended on a horizontal rod (5 × 5 mm area, 35 cm long, between two 50 cm high poles), and the suspension latencies were measured for 5 min.

#### 4.4.4. Y-Maze Test

The Y-maze test is used to evaluate cognition and short-term spatial memory [79]. This test was carried out in an enclosed “Y” shaped maze (Jeung Do B&P, Seoul, Korea). Normal mice tend to visit the arms of the maze one after the other. This behavior is called spontaneous alteration and is used to assess short-term spatial memory in a new environment. The number of each arm entries, spontaneous alteration, and percent alteration were recorded and determined for 8 min. The percent alteration was calculated as follows: [number of spontaneous alteration/(number of total arm entries − 2)] × 100. At the end of each trial, the maze was cleaned of urine and feces with 70% ethanol.

### 4.5. Molecular Assessments

#### 4.5.1. Quantitative Real-Time PCR (qRT-PCR)

Total RNA was prepared in the studied brain tissue lysates using a TRIzol reagent (Invitrogen Life Technologies, Carlsbad, CA, USA), according to the manufacturer’s instructions. A nanodrop spectrophotometer (Thermo Fisher Scientific, Waltham, MA, USA) was used to confirm the quality and quantity of extracted RNA. Differentially expressed genes of interest related to presynaptic scaffold proteins from the cerebral cortex and hippocampus were selected to be validated by a qRT-PCR. A ReverTra Ace^®^ qPCR RT Master Mix with gDNA Remover (Toyobo, Osaka, Japan) was used to synthesize cDNA with total RNA. Then, 2 μL of cDNA in a total volume of 20 μL was used in the following reaction. The qRT-PCR was performed in triplicate on a Light Cycler 480 (Roche Applied Science, Mannheim, Germany), using the Light Cycler 480 SYBR Green master mix (Roche), with thermocycler conditions as follows: amplifications were performed starting with a 300 s template preincubation step at 95 °C, followed by 45 cycles at 95 °C for 10 s, 60 °C for 10 s, and 72 °C for 10 s. The melting curve analysis began at 95 °C for 5 s, followed by 1 min at 60 °C. The specificity of the produced amplification product was confirmed by the examination of a melting curve analysis, and showed a distinct single sharp peak with the expected Tm for all samples. A distinct single peak indicates that a single DNA sequence was amplified during the qRT-PCR. The detail sequence of the primers is listed in Table S1. Primers were designed using the NCBI primer blast, with the parameters set to a product of 150–200 bp within the region surrounding the identified translocation. The expression of each gene of interest was obtained using the 2^−ΔΔ*C*t^ method. The expression level of each gene of interest was obtained using the 2^−ΔΔ*C*t^ method. Target-gene expression was normalized relative to the expression of GAPDH and represented as fold change relative to the control.

#### 4.5.2. Western Blot

To confirm the expression of Ca_v_ 2.1 and synaptic proteins in the cerebral cortex and hippocampus in the EE and control mice, 30 μg of total protein was extracted from all mice and dissolved in a sample buffer (60 mM Tris–HCl, pH 6.8, 14.4 mM b-mercaptoethanol, 25% glycerol, 2% SDS, and 0.1% bromophenol blue; Invitrogen), incubated for 10 min at 70 °C, and separated on a 10% SDS reducing polyacrylamide gel (Invitrogen). Protein samples were separated with SDS-polyacrylamide gel electrophoresis (PAGE) on a 4–12% gradient Bis-Tris gel and Tris-Acetate gel (Invitrogen, Carlsbad, CA, USA). The separated proteins were further transferred onto a 0.45 μm invitrolonTM polyvinylidene difluoride (PVDF) filter paper sandwich using an XCell IITM Blot Module (invitrogen, Life Technologies, Carlsbad, CA, USA). The membranes were blocked for one hour in Tris-buffered saline (TBS) (10 mM Tris-HCl, pH 7.5, 150 mM NaCl) plus 0.05% Tween 20 (TBST) containing 5% non-fat dry milk (Bio-Rad, Hercules, CA, USA) at room temperature, washed three times with TBST, and incubated at 4 °C overnight with the following primary antibodies; anti-Munc13 (1:1000, Abcam), anti-Raphilin3A (1:1000, Synaptic Systems), anti-Munc18 (1:1000, Abcam), anti-VAMP2 (1:1000, Abcam), anti-SNAP25 (1:1000, Abcam), anti-Syntaxin (1:1000, Abcam), anti-Ca_v_ 2.1 (1:1000, Abcam), and anti-ACTIN (1:5000, Santa Cruz). After washing the blots three times with TBST, the blots were incubated for one hour with horseradish peroxidase-conjugated secondary antibodies (1:5000; Santa Cruz, CA, USA) at room temperature. The proteins were further washed three times with TBST and visualized with an enhanced chemiluminescence (ECL) detection system (Amersham Pharmacia Biotech, Little Chalfont, UK). Using ImageQuant™ LAS 4000 software (GE Healthcare Life Science, Chicago, IL, USA), Western blot results were saved into TIFF image files, and then the images and the density of the band were analyzed and expressed as the ratio relative to the control band density using Multi-Gauge (Fuji Photo Film, version 3.0, Tokyo, Japan). To normalize the values of all samples to account for band intensity, the average band intensity for each mouse group was first calculated. The samples were normalized to the group average of controls, and target protein expressions were normalized relative to the expression of ACTIN. The value of the control group was set to 1 and was divided by the value of each individual mouse.

### 4.6. Immunohistochemistry

The brain tissues were frozen in Surgipath FSC 22 clear frozen section compound (Leica Microsystems, Wetzlar, Germany) using dry ice and isopentane. The harvested brain tissues were cryosectioned at 16-μm thickness along the coronal plane, and immunohistochemistry staining was performed. At 8 weeks after EE, to confirm the endogenous expression of Cav 2.1 (1:100, Abcam) and MAP2 (1:400, Millipore, Burlington, MA, USA), the brain sections of the cerebral cortex, hippocampus, and striatum were immunostained. The sections were incubated with Alexa Fluor^®^ 488 goat anti-rabbit (1:400, Invitrogen) and Alexa Fluor^®^ 594 goat anti-mouse (1:400, Invitrogen) secondary antibodies, then covered with Vectashield^®^ mounting medium with 4C, 6-diamidino-2-phenylindole (DAPI; Vector, Burlingame, CA, USA). The stained sections were analyzed using confocal microscopy (LSM700; Zeiss, Gottingen, Germany).

### 4.7. Statistical Analysis

Statistical analyses were performed using Statistical Package for Social Sciences software version 25.0 (IBM Corporation, Armonk, NY, USA). The continuous variables of molecular and histological assessments were compared between groups by a Mann–Whitney U test. A *p* value < 0.05 was considered statistically significant. A two-way repeated measure analysis of variance (ANOVA) test was used to examine the main and interaction effects within and between groups (5 × 3 factorial design) for the rotarod test. Post hoc analysis was used to find where the significant differences were, and was identified at *p*-value of <0.01 using a Bonferroni adjustment as a multiple pairwise comparison. For comparison among the three experimental groups in the other behavioral assessments, one-way ANOVA with least significant difference (LSD) for post-hoc comparison was conducted. All graphical artworks were produced using GraphPad Prism version 8.4.3 (GraphPad Software lnc., San Diego, CA, USA).

## 5. Conclusions

In this study, we have shown that EE improves cognitive and motor functions in mice with chronic HI brain injuries that mimic the pathophysiology of human HIE. These beneficial effects of EE may be due to the increased expression of Cav 2.1 in neurons and the upregulation of presynaptic proteins that are related to the synaptic vesicle cycle and neurotransmitter release in the cerebral cortex and hippocampus, which, in turn, may contribute to behavior improvement. 

## Data Availability

Data is contained within the article or Supplementary Material. The data presented in this study are available.

## Supplementary Materials

The following are available online at https://www.mdpi.com/article/10.3390/ijms22073414/s1, Table S1: List of primers used for qRT-PCR quantification, Figure S1: EE upregulates the expression of Ca_v_ 2.1 and induces higher colocalization of Ca_v_2.1 with MAP2 in striatum in HIE mice, Figure S2: The higher colocalization with Ca_v_ 2.1 is noticed in neuron-related markers in HI EE mice.
