# Supplementary material for: Environmental Enrichment Enhances Cav 2.1 Channel-Mediated Presynaptic Plasticity in Hypoxic–Ischemic Encephalopathy
Source: Int J Mol Sci. 2021 Mar 26;22(7):3414. doi: 10.3390/ijms22073414 (PMC8037860; doi:10.3390/ijms22073414)
Supplement: Supplementary file 1 [file ijms-22-03414-s001.zip › Supplementary Figures.pdf]

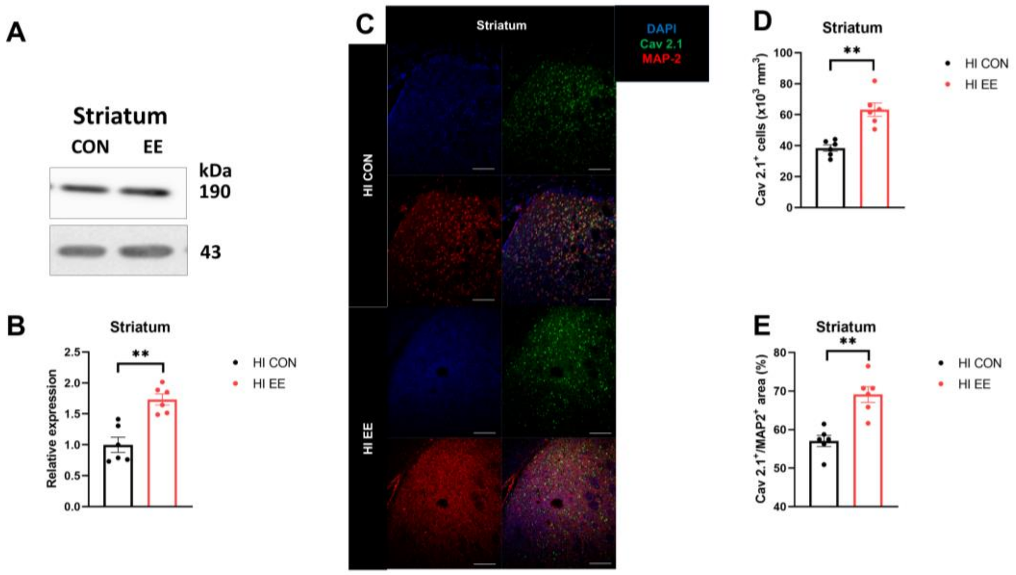

Figure S1. EE upregulates the expression of Ca_v 2.1 and induces higher colocalization of Ca_v2.1 with MAP2 in striatum in HIE mice (**A**) The representative WB images of Ca_v 2.1 in striatum. (**B**) Quantification of Ca_v 2.1 protein expression in striatum. Significant difference was observed between HI control mice and HI EE mice in striatum. (**C**) The representative confocal images of Ca_v 2.1 and MAP2 in striatum. A white bar is 100 µm. (**D**) The number of Ca_v 2.1^+ cells in striatum was significantly different between HI control mice and HI EE mice. (**E**) Significant difference in the area of Ca_v 2.1^+ $MAP2^+$ was observed between HI control mice and HI EE mice in striatum. Molecular and histological data are expressed as mean ± SEM with 6 mice per group (**P<0.01, Mann-Whitney U test).

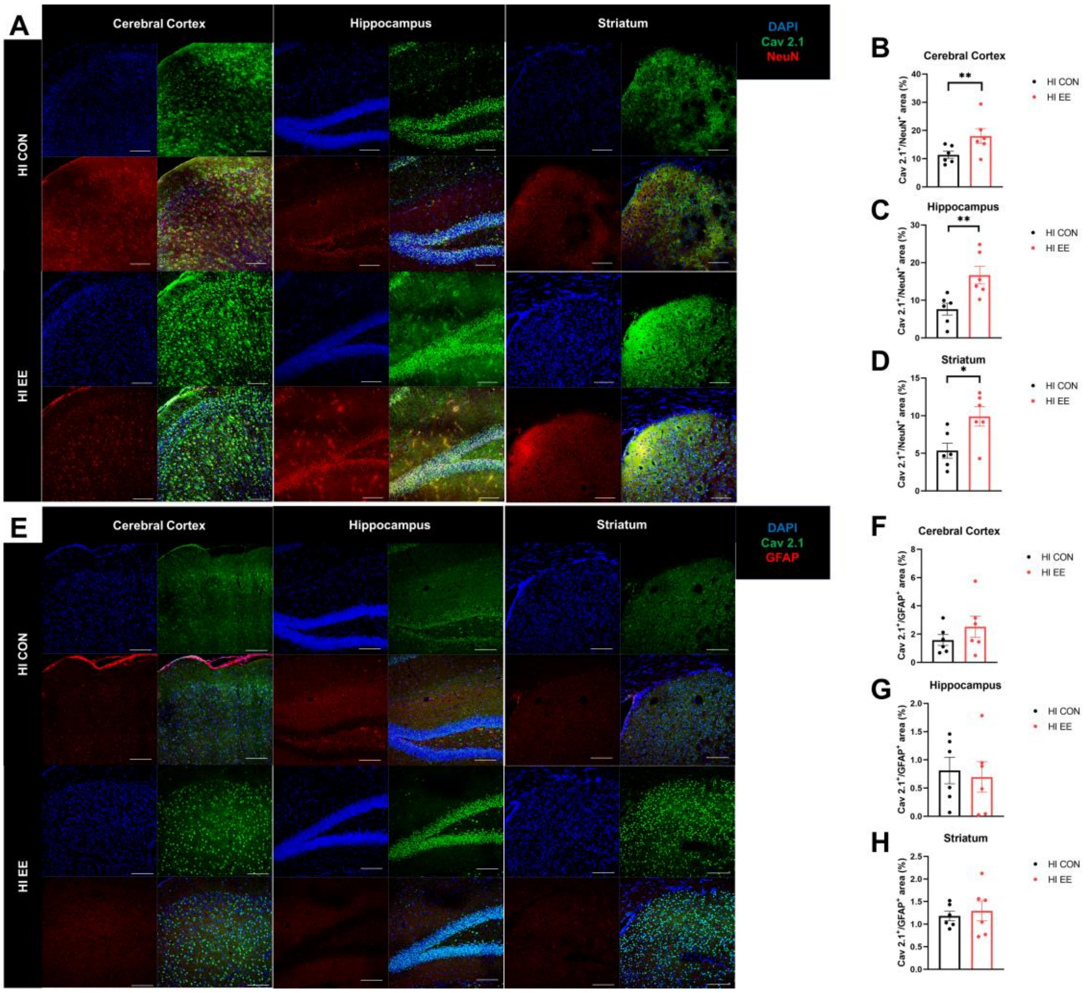

Figure S2. The higher colocalization with Ca_v 2.1 is noticed in neuron-related markers in HI EE mice. (**A**) The representative confocal images of Ca_v 2.1 and NeuN in cerebral cortex, hippocampus, and striatum. (**B**-**D**) Significant difference in the area of Ca_v 2.1^{+}NeuN^{+} was observed between HI control mice and HI EE mice in cerebral cortex, hippocampus, and striatum, respectively. (**E**) The representative confocal images of Ca_v 2.1 and GFAP in cerebral cortex, hippocampus, and striatum. Relative to the neuronal markers such as MAP2 and NeuN, the small subpopulation of GFAP, an astrocyte marker, was colocalized with Ca_v 2.1. (**F**-**H**) No significant difference in the area of Ca_v 2.1^{+}GFAP^{+} was observed between HI control mice and HI EE mice in cerebral cortex, hippocampus, and striatum, respectively. Histological data are expressed as mean ± SEM with 6 mice per group (*$P<0.05$, **$P<0.01$, Mann-Whitney U test). A white bar is 100 μm.
