# Supplementary material for: Environmental Enrichment Enhances Cav 2.1 Channel-Mediated Presynaptic Plasticity in Hypoxic–Ischemic Encephalopathy
Source: Int J Mol Sci. 2021 Mar 26;22(7):3414. doi: 10.3390/ijms22073414 (PMC8037860; doi:10.3390/ijms22073414)
Supplement: Supplementary file 1 [file ijms-22-03414-s001.zip › Supplementary Table.pdf]

Supplementary Table S1. List of primers used for qRT-PCR quantification

Gene	Forward primer	Reverse primer
RIM	CAAACGTGTCCCCAAGTCTGT	CAGTACCTAGTCTGATCCTC
Munc13	GCTCACAGAGTCGTTCCTCC	GGTGGTCTGCCAGTTTAGGT
Raphilin3A	CCAAGACAACAGCAACCTGC	CCAAGACAACAGCAACCTGC
Munc18	CGGTCCCCGCCTCATTATTT	CTGAAGTCGGGGTGCTCAG
VAMP2	GAGCGGGACCAGAAGTTGTC	GCGCAGATCACTCCCAAGATG
SNAP25	GGATGAGCAAGGCGAACAAC	TCCTGATTATTGCCCCAGGC
Syntaxin	AGGATCGGACTCAGGAGCTG	CTCACCCTGGCTCTCTACGA
CACNA1A	CACGACACACAGACAGTTCC	AGCGGGCGAGTAGGACAC
GAPDH	CATCACTGCCACCCAGAAGACTG	ATGCCAGTGAGCTTCCCGTTCAG

GAPDH: Glyceraldehyde 3-phosphate dehydrogenase was used as an internal control; CACNA1A: The gene name of Ca_v 2.1 channel.
